# ARE STEM CELL MARKER EXPRESSION AND CD133 ANALYSIS RELEVANT TO DIFFERENTIATE COLORECTAL CANCER?

**DOI:** 10.1590/0102-672020210002e1585

**Published:** 2021-10-18

**Authors:** Leticia Elizabeth Augustin CZECZKO, Carmen Australia Paredes Marcondes RIBAS, Nicolau Gregori CZECZKO, Thelma Larocca SKARE, Camila Kienen YAMAKAWA, Guilherme GIONEDIS, Cecilia VASCONCELOS, Fabiola Pabst BREMER, Diogo Francesco CASTOLDI, Martin GASSER, Ana Maria WAAGA-GASSER

**Affiliations:** 1 Mackenzie Evangelical Faculty of Paraná, Curitiba, PR, Brazil; 2 University Evangelical Mackenzie Hospital, Curitiba, PR, Brazil; 3 Renal Division, Brigham and Woman’s Hospital, Harvard Medical School, Boston, MA, USA

**Keywords:** Colorectal neoplasms, Adenoma, Biomarkers, tumor, Proto-oncogene proteins c-MYC, AC133 antigen, Receptor protein tyrosine-kinase, Neoplasias colorretais, Adenoma, Biomarcadores tumorais, Proteínas proto-oncogênicas, C-myc, Antígeno ac133, Receptores proteína tirosina quinases

## Abstract

***Background*:**

CD133 and AXL have been described as cancer stem cell markers, and c-MYC as a key regulatory cellular mechanism in colorectal cancer (CRC).

***Aim*:**

Evaluate the prognostic role of the biomarkers CD133, AXL and c-MYC and their association with clinicopathologic characteristics in colorectal adenocarcinomas and adenomas.

***Methods*:**

A total of 156 patients with UICC stage I-IV adenocarcinomas (n=122) and adenomas (n=34) were analyzed. Tissue microarrays (TMA) from primary tumors and polyps for CD133, c-MYC and AXL expression were performed and analyzed for their significance with clinicopathologic characteristics.

***Results*:**

Poorly differentiated adenocarcinomas and disease progression were independent risk factors for poor overall survival. The median overall survival time was 30 months. Positive CD133 expression (35.9% of all cases), particularly of right-sided CRCs (44.8% of the CD133+ cases), was negatively correlated with death in the univariate analysis, which did not reach significance in the multivariate analysis. c-MYC (15.4% of all cases) was predominantly expressed in advanced-stage patients with distant (non-pulmonary/non-hepatic) metastasis. AXL expression was found only occasionally, and predominantly dominated in adenomas, with less penetrance in high-grade dysplasia.

***Conclusions*:**

CD133 expression was not associated with inferior overall survival in CRC. While AXL showed inconclusive results, c-MYC expression in primary CRCs was associated with distant metastasis.

## RESUMO

**Racional:**

CD133 e AXL são descritos na literatura como marcadores de células-tronco tumorais, e c-MYC cumpre papel chave como mecanismo de regulação celular no câncer colorretal (CCR).

**Objetivo:**

Avaliar o papel prognóstico dos biomarcadores CD133, AXL e c-MYC e sua associação com características clinicopatológicas de adenocarcinomas e adenomas colorretais.

**Métodos:**

Um total de 156 pacientes com adenocarcinomas de estádio UICC I-IV (n=122) e adenomas (n=34) colorretais foram avaliados. Microarranjos teciduais (TMA) dos tumores primários e adenomas foram realizados em busca de expressão de CD133, c-MYC e AXL, com posterior análise de relação significativa com características clinicopatológicas.

**Resultados:**

Adenocarcinomas pobremente diferenciados e progressão de doença foram fatores de risco independentes para má sobrevida global. A taxa mediana de sobrevida global foi de 30 meses. Expressão positiva de CD133 (35,9% dos casos), particularmente em cânceres de cólon direito (44,8% dos casos CD133+), correlacionou-se negativamente com óbito na análise univariada, sem significância estatística na análise multivariada. c-MYC (15,4% dos casos) teve predomínio de expressão em pacientes com estádio avançado com metástases distantes (não-pulmonares/não-hepáticas). Expressão de AXL foi pouco encontrada, com predomínio em adenomas, com menor penetrância em displasia de alto grau.

**Conclusão:**

Expressão de CD133 não se associou com sobrevida global inferior em CCR. Enquanto AXL demonstrou resultados inconclusivos, expressão de c-MYC em tumores primários se associou-se à metástases à distância.

## INTRODUCTION

Colorectal cancer (CRC) is one of the major malignancies in humans and the second and third leading cause of cancer-related deaths in men and women in the United States and Brazil, respectively^17, 22^. Molecular pathways in colorectal carcinogenesis have increasingly been characterized in the past two decades. The proto-oncogene KRAS was the first gene that became integrated into clinical decision-making process for additional biological therapy in advanced stage CRC, as well as in other types of cancer, within the last 15 years. Subsequently, dividing CRC patients into further defined subgroups, with either high or low risk of progression depending on current molecular findings, is becoming more common in clinical care, as it may lead to several therapeutic implications. The Cancer Genome Atlas revealed that CRC contains well known genes with significant mutation, such as APC, TP53, SMAD4, PIK3CA and KRAS, and others such as ARID1A, SOX9 and FAM123B. Approximately 16% of analyzed CRCs demonstrated a high mutational load, and 75% out of those had high microsatellite instability (MSI-H), usually with hypermethylation and silencing of the MLH1 gene. Moreover, 25% of the cases had somatic mutations in the mismatch-repair (MMR) and POLE genes (ε polymerase)^15^. 

Colorectal adenomas are premalignant lesions of CRC with increased proliferative capacity and accumulation of pathologic molecular patterns that become distinctly detectable during progression, depending on the timeline of each adenoma and its histological subgroup^2,9,17.^ Better characterization of molecular pathways that occur during the adenoma-to-adenocarcinoma sequence and during disease progression, from early to advanced stages, is of great relevance for better understanding of CRC pathophysiology and its development process^7^. 

An increasing number of publications have described so called biomarkers that may allow to better define prognostic data in individual patients with or without mutations in specific proto-oncogenes (like KRAS) and tumor suppressor genes (like TP53) in colorectal tumor cells, but there is still a lot of controversy regarding clinicopathological outcomes.

Recently, it has been consistently suggested that many of these mutational findings in oncogenes and suppressor genes may be of specific relevance in tumor cells with pluripotent characteristics and may therefore predominantly drive tumor progression. Such cells were defined to be derived from cancer stem cells (CSC) located within the intestinal crypts during initial colorectal carcinogenesis. In contrast to the bulk of terminally differentiated tumor cells, this small group of progenitor cells can sustain their key characteristics of self-renewal and differentiation as they develop into colorectal tumor cells^26^

There is growing evidence that carcinogenesis in CRC is driven particularly by this small group of pluripotent cancer cells, which may harbor upregulated proto-oncogenes and growth regulatory mechanisms and therefore may become relevant in tumor progression. CD133, also called Prominin-1 is one of the most frequently described transmembrane glycoproteins most frequently associated with progenitor cell characteristics and stem cell behavior in CRC and is currently considered the most robust surface marker for CRC progenitor cells^12^.

The receptor tyrosine kinase AXL regulates several vital cellular processes, including proliferation, survival, motility and immune response. Although it is not implicated as an oncogenic driver, AXL is overexpressed in several hematologic and solid malignancies, such as acute myeloid leukemia, non-small-cell lung cancer, gastric and colorectal adenocarcinoma, as well as prostate and breast cancer^10^. c-MYC is one of the main genes responsible for growth regulation and cellular metabolism. Its overexpression turns the c-MYC proto-oncogene into a potent oncogene responsible for supplying the necessary metabolism for fast cellular proliferation and coordinating the changes in gene families that increase cellular proliferation^19^.

The aim of the present study was to evaluate the prognostic role of the biomarkers CD133, AXL and c-MYC and their association with clinicopathologic characteristics in colorectal adenocarcinomas and adenomas, in a single institutional patient outcome.

## METHODS

### Patients and tissue samples

This retrospective observational study was approved by the ethics committee of the Mackenzie Evangelical Faculty of Paraná, Curitiba, PR, Brazil, (IRB approval number: 1999672) and was divided into two distinct groups: the adenocarcinoma group (n=122) included patients with colorectal cancer diagnosed between 01/2010 and 12/2015; and the adenoma group (n=39) included patients with colorectal adenomas who underwent polypectomy performed through colonoscopy between 01/2009 and 12/2012. All cases were recruited from the University Evangelical Mackenzie Hospital, Curitiba, PR, Brazil. The paraffin blocks and histopathology slides of the CRCs and adenomas were obtained and referred for a histological review before the immunohistochemistry assay. 

### Multisampling block construction and immunohistochemistry (TMA)

This study used the tissue microarray technique (TMA) for histological analysis. First, the H&E-stained slides were analyzed and the area containing the largest representation of neoplasia was marked with a pen. The corresponding paraffin donor block received the same mark in the same place of the slide. The selected area was extracted from the donor blocks with the Tissue-Tek Quick-Ray^TM^ (Sakura, Nagano, Japan) device, extracting 2.0 mm cylindrical tissue core “biopsies”. An Excel table with 10 columns and six rows was created to coordinate the array. Each of the 60 cylindrical tissues were placed in the corresponding holes in the paraffin mold, following the Excel order, and then the mold was filled with paraffin, finalizing the TMA block. A 5 µm multiple sections microtomy was made in each TMA block on Thermo Scientific^TM^ Superfrost Plus^TM^ (Thermo Fisher Scientific, Waltham, Massachusetts, United States) hydrophilic slides. 

The cases were submitted to the immunoperoxidase technique, performed in the instrument BenchMark ULTRA^TM^ (Roche Tissue Diagnostics, Basel, Switzerland), with integrated 3-in-1 processing. Reading was performed through an amplifier after the staining of primary antibodies. Two pathologists made their reports on the slides at different times and the samples were classified as positive, in case they presented the staining by the antibody, or negative otherwise. The following antibodies were used: CD133 polyclonal (Biorbyt, Cambridge, United Kingdom); AXL polyclonal (St John’s Laboratory Ltd., London, United Kingdom); c-MYC clone MYC275 + MYC909 (Medaysis, Livermore, United States).

### Data collection

Clinicopathological data was obtained using the hospital’s electronic clinical database (PAGU), chemotherapy requirement forms (APACs) and official anatomopathological reports. In order to collect additional information, phone contact was also made. Each case was inserted in a standardized data collection protocol. The adenocarcinoma group protocol contained patient pseudonym with initials, age at diagnosis, gender, phone number, paraffin block number, date of surgery, histological diagnosis, primary organ tumor site, ICD-10, primary case, pathological TNM, distant metastasis site (if any), UICC staging, resection status, progression date (if any), disease free survival (if any), date of progression (if any), progression of distant metastasis (if any), overall survival, follow-up and date when ‘last seen’. 

Likewise, the adenoma group protocol had pseudonym with initials, age at diagnosis, gender, paraffin block number, date of colonoscopy, adenoma location, adenoma macroscopic classification, histological diagnosis, size at colonoscopy, and grade of dysplasia.

### Statistical analysis

All data were analyzed with the computing program Stata/SE v.14.1 (Stata Corp Lp., College Station, Texas, United States). To analyze factors associated with the progression event (PEVENT), Fine and Gray models were used, considering death as a competitive risk. After adjustments, the estimated association measure used was the sub distribution hazard ratio (SHR). To analyze survival, univariate and multivariate-adjusted Cox regression models were used, and the hazard ratio (HR) values were estimated. To assess the significance of each variable, the Wald test was used. Values of p<0,05 indicated statistical significance. 

## RESULTS

### Patient outcomes

The clinicopathologic variables of the adenocarcinoma group (n=122) are described in Table 1. 

**TABLE 1:** Clinical-pathological variables of the adenocarcinoma group and CD133+ and c-MYC+ (positive staining). The AXL analysis was not performed (AXL+ =4)

	n	CD133+	p value	c-MYC+	p value
Gender			p=0.52		p=0.53
Male	63	14		34	
Female	59	17		30	
Age (years)			p=0.26		p=0.20
< 50	22	7		11	
50 a 65	45	14		41	
>65	55	10		12	
Primary organ tumor site			p=0.19		p=0.17
Right colon	42	13		18	
Left colon	48	10		24	
Rectum	27	6		19	
UICC staging			p=0.51		p=0.67
0/I/II	41	14		25	
III/IV	62	17		39	
Histologic grade			p=0.42		
Low/moderately	93	27		60	
High	7	3		4	
Pathological TNM staging					
pTins/pT2	13	3	p=0.74	7	p=0.37
pT3/pT4	84	27		53	
pN0	44	14	p=1	27	p=1
pN1/pN2	48	15		29	
pM0	11	2	P=0.69	9	p=1
pM1	28	8		18	
Distant metastasis site					
Pulmonar	4	2	p=0.58	1	p=0.14
Hepatic	21	5	p=0.59	15	p=0.30
Peritoneal	8	0	p=0.10	7	p=0.25
Other	8	1	p=0.43	7	p=0.04

**FIGURE 1:**
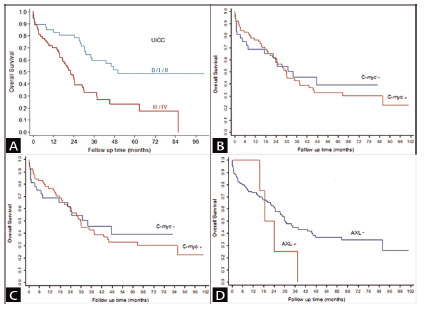
Kaplan-Meier curve: A) according to early UICC staging (0/I/II) and late UICC staging (III/IV); B) comparing CD133+ and CD133- tumors; C) comparing c-MYC+ and c-MYC- tumors; D) comparing AXL+ and AXL- tumors

The most common distant metastasis site was the liver, with 24 cases (19,7%). The overall survival rate was 44.3% and the median overall survival time estimated by the Kaplan-Meier method was 30 months. The univariate analysis demonstrated that early stage adenocarcinomas were associated with better overall survival (Figure 1A). It was observed that poorly differentiated adenocarcinomas and disease progression were independent risk factors for death (p<0.05). Positive CD133 expression (Figure 1B) was negatively correlated with death in the univariate analysis, but this relevance was lost in the multivariate analysis. c-MYC and AXL expressions were not associated with overall survival (Figures 1C and 1D).

There was no statistically significant association between CD133 expression and age, gender, primary tumor location, presence of distant metastasis, histologic grade, tumor size, presence of lymph node metastasis and UICC staging. 

**FIGURE 2:**
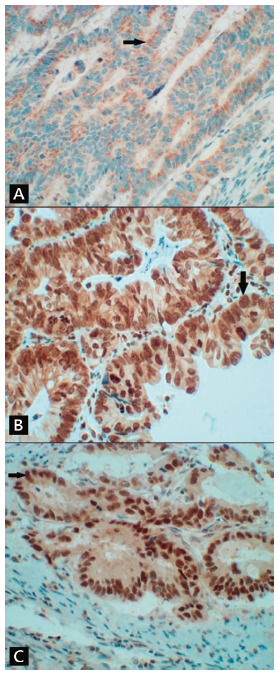
Colorectal adenocarcinoma photomicrograph: A) CD133+ showing cytoplasmic positivity (arrow): B) AXL+ showing combined positivity (cytoplasmic membrane, cytoplasm and nuclear - arrow); C) c-MYC+ showing nuclear positivity (arrow, 400x)

Out of 122 patients, four individuals presented with positive staining for AXL (AXL+, Figure 2B), 97 negative (AXL-) and 21 were inconclusive. The statistical analysis was unable to be concluded due to the low number of AXL+ cases.

### Positive c-MYC expression was associated with distant metastasis

Out of 122 patients, 64 presented with positive staining for c-MYC (c-MYC+, Figure 1C), 38 were negative (c-MYC-) and 20 cases were deemed inconclusive. 

Eleven patients presented distant metastasis to “other sites” (excluding liver, lung and peritoneum). Out such cases, 63.6% (n=7) were c-MYC positive, with p<0.05. This outcome showed a positive association between c-MYC expression and the presence of distant metastasis. There was no statistically significant association between the c-MYC status and age, gender, primary tumor location, presence of distant metastasis, histologic grade, tumor size, presence of lymph node metastasis and UICC staging.

### Colorectal adenomas demonstrated CD133, c-MYC and AXL expression with no association to clinicopathologic characteristics

The analysis performed in the adenoma group was based on the data from 34 patients and a total of 39 adenomas. The group consisted of 16 males and 18 females. Patient age ranged from 34 to 96 years old, with an average age of 67.8 years. The clinicopathological characteristics of the adenoma group are described in Tables 2 and 3. 

**TABLE 2:** Clinical and pathological characteristics of the polyp group (n=39) and CD133+, c-MYc+ and AXL+ (positive staining) with the percentage of each variable considering positive, negative and undetermined immunostaining results (p>0,05)

Variables	n	CD133+	c-MYC+	AXL+
Anatomic Location				
Ascending/ transverse colon	9	5 (55%)	1 (20%)	1 (25%)
Descending colon	6	2 (20%)	1 (20%)	1 (20%)
Sigmoid	17	6 (35.3%)	3 (17.7%)	4 (23.5%)
Rectum/anus	6	2 (33%)	1 (20%)	
Type				
Plane	2	1		1
Sessile	11	4 (22.2%)		
Semipedunculated	5	1 (20%)	1 (20%)	1 (20%)
Pedunculated	18	7(38.9%)	5 (27.8%)	4 (22.2%)
Undetermined	3	1(33.3%)		
Histologic diagnosis				
Adenoma	31	13(41.9%)	6(19.4%)	
Hyperplasic polyp	6	1 (16.7%)		
Adenocarcinoma	1			
Inflammatory polyp	1			
Displasia				
Absent	23	6 (26.1%)	3 (13%)	2 (8.7%)
Low	0			
High	15	8 (57.1%)	2 (14.3%)	3 (21.4%)
Adenocarcinoma in situ	1			

**TABLE 3:** Immunostaining results of the CD133, c-MYC and AXL

	CD133	c-MYC	AXL
Positive	14 (35.9%)	6 (15.4%),	6 (15.4%)
Negative	10 (25.6%)	17 (43.6%)	20 (51.3%)
Inconclusive	15 (38.5%)	16 (41%)	13 (33.3%)

In the univariate analysis, there was no statistical significance between CD133+, c-MYC+ and AXL+ adenomas and size, anatomic location, type, histological diagnosis and status of dysplasia.

### Agreement between markers

In the adenocarcinoma group, a moderate agreement between c-MYC and AXL was found using the Kappa coefficient of agreement, since it was seen 28 (71.8%) concordant cases (c-MYC+/AXL+ or c-MYC- /AXL-) and 11 (28.2%) discordant cases.

## DISCUSSION

As already referred, colorectal cancer is the third most common cancer in Brazil and in the world^22^. In order to improve the understanding of its molecular pathophysiology, several studies regarding different genetic and proteic expressions have been published throughout the years.

Out of all molecular techniques that analyze mutations in cancer cells, immunohistochemistry is one of the widely utilized and most accessible to be performed. In the last two decades, tissue microarrays (TMAs) have been used as the technique of choice in several immunohistochemical analysis studies, since it drastically diminishes costs and other resources as well as reduces tissue wasting^28^. 

In the present study, the expression of three different biomarkers in 156 patients was analyzed using the TMA method. Instead of performing individual immunohistochemical sections and stainings on 156 paraffin-embedded tumor blocks, small cores from each sample with the best representative area were arrayed onto a small number of TMA blocks. However, in colorectal cancer, tissue heterogeneity seems particularly pronounced, and therefore small biopsies and tissue sections have an intrinsic risk of sampling error when said tissue is tested for therapy-related biomarkers^3^.

To evaluate this possible bias, a study was performed with seven immunomarkers in 44 breast carcinoma samples. Intratumoral variance was seen in five immunomarkers. This indicated the problematic interpretation of small biopsy specimens as being representative for the status of the entire tumor^15^. In another study regarding colorectal cancer, a TMA was performed using tissues of 340 patients. Eight different tissue spots were taken from as many different cancer blocks per patient as possible. Immunohistochemical analysis was performed for HER2 and p53, and while 93.2% of the samples showed a homogenous distribution for p53, 88.9% of the samples had heterogeneous results for HER2^16^. As a result, further studies in CRC using ’heterogeneity TMAs‘ are demanded to prove its underlying significance, with the inclusion of more than only one representative tumor fragment for a more consistent result about tumor molecular mutations and related protein expression.

 The goal of studying cancer and pre-neoplastic lesions, such as colorectal adenomas, is to have a database for each type and subtype of tumor, a specific ‘tumor molecular diagnosis’. With the immunohistochemical technique, it is possible to isolate the tumor types that need greater attention in relation to follow-up and, putatively, higher intensity of additive chemotherapy and/or biological therapy. It is also important to understand which are the genes and pathways that are hyperactivated or suppressed, and therefore attempt to molecularly correct the triggering factors with emerging treatment strategies, such as targeted therapy.

The recent available literature still has great controversy between the results of colorectal tumor biomarkers particularly in cancer stem cell. This association of poor prognosis had already been described by other groups^25^. In order to find a definite conclusion, a meta-analysis was published including 37 articles about CD133 expression, determining that CRC cases with a positive CD133 expression have more aggressive clinicopathological characteristics and worse prognosis^11^. Although CD133 has been used as a cancer stem cells marker, little is known about its function. Whether CD133 participates in the biological behavior of CSC or merely acts as a marker of the CSC phenotype is not clear. Two studies were performed to investigate the types of genomic instability and cancer stem cells in colorectal cancer, and both found that high levels of CD133 expression is associated with microsatellite-stable colorectal cancer^5^ ^,^ ^18^.

This study shows a significant association between adenocarcinomas with c-MYC expression and the presence of distant metastasis (p<0.05). However, opposite outcomes also exist for this relevant transcription factor in the literature, reporting that patients with MYC positive tumors demonstrated a better 5-year survival^23^. Trying to justify the difference of significance observed in studies involving immunohistochemistry of c-MYC, a study was performed using two types of MYC antibodies: N-terminal (Y69) and C-terminal (9E10), with posterior association to ISH (in situ hybridization) results. The authors concluded that the antibodies that target the C-terminal end (9E10) needed to be interpreted cautiously^1^. 

Further molecular biologic methods were used to define the expression of the c-MYC oncogene. A study utilized the SISH (silver in situ hybridization) and ddPCR (droplet digital polymerase chain reaction) were used to detect the presence of the c-MYC gene in CRC, and concluded that c-MYC overexpression is an independent factor of worse prognosis (p=0.002)^13^. Other bioinformatic studies analyzed and published in the Cancer Genome Atlas aiming to identify the prognostic role of biomarkers and possible targeted therapies for CRC concluded that MYC influences several tumor pathways and can be used as a predicting candidate for worse prognosis and targeted therapy^8^. 

The present study showed a very low rate of AXL-positive adenocarcinomas, making it not possible to perform any statistical analysis. Nevertheless, AXL still holds controversy regarding its use as a biomarker and for targeted therapy. A recent study demonstrated the paradoxical effect of AXL and the receptor tyrosine kinase Mer in colon cancer. The authors stated that AXL and Mer are expressed in several tumor cells and have their oncogenic role well established, but their gene silencing led to an increase of post-inflammatory cytokines, favoring a tumor-promoting environment^4^. Another study with 223 patients showed AXL-positive tumors in 76.7% of the samples, and such tumors were correlated with less differentiated tumors (p<0,05). The administration of AXL inhibitors in orthotopic colon cancer models using HCT116 tumor cells resulted in significant inhibition of tumor growth and peritoneal metastatic dissemination. The authors concluded from their results that the inhibition of AXL can represent a new therapeutic approach for colorectal cancer^14^. 

Another study including 18 cases of CRC with subsequent silencing of AXL in a panel of colorectal cancer cell lines showed a statistically significant correlation between AXL expression and stage IV cancers in comparison to stage 0. Moreover, gene silencing significantly inhibited the migration and invasion of tumor cells but did not reduce the proliferation and survival of tumor cells, as well as did not stimulate the increase of apoptosis and chemosensitivity. However, controversial results were seen between studies regarding the relevance of AXL expression in colorectal cancer cells HCT66^24^.

In the adenoma group, no statistically significant expression or correlation with patient outcome data were found for the biomarkers CD133, c-MYC and AXL. Such results seem to suggest that the expression of these tumor biomarkers is not of significant importance in non-malignant colorectal tissues based on TMA technique. Two studies comprising 243 cases evaluated the presence of CD133 in colorectal adenomas using the two different methods - immunohistochemistry and immunohistochemistry with microarray, and both emphasized the importance of CD133 for colorectal carcinogenesis^12^ ^,^ ^27^. The c-MYC oncogene was studied in a multiomics analysis of 275 patients pairing their normal colon tissue to the neoplastic tissue (from adenomas to UICC stage IV adenocarcinomas). The authors concluded that global metabolic reprogramming of CRC occurs at the adenoma stage and is induced by MYC^19^. 

Another immunohistochemical study was performed in 380 adenomas for differences in molecular Ki-67, COX-2, TGFβRI, EGFR, β-catenin, cyclin D1, c-MYC and TUNEL (apoptosis) mutations of proximal and distal adenomas, which may contribute for cancer heterogeneity between the two topographies. The authors concluded that the adenoma location is not one of the main determiners of expression of the analyzed biomarkers outside of other pathological features^21^. Another research evaluated the expression of 29 markers of 50 patients pairing their neoplastic tissue (from adenoma to hepatic metastasis) with a normal mucosa sample. The authors concluded that the tyrosine kinase Arg has a possible role in colon carcinogenesis, especially in the transition from adenoma to carcinoma^6^.

 There are several reasons for divergences in studies of tumor biomarkers. Different techniques directly related to the immunohistochemistry reaction and procedure, the quality of the antibodies, the absence of standard protocols for positive and negative controls, different chemical fixation methods, and also the quantity and quality of material and the type of specimens to be analyzed (fresh or fixed in formaldehyde). Among the analyzed studies, there is no methodological standardization that would make the outcome more reliable for comparison. Some examples of such problem are different antibodies for the same biomarker, the variability of the concept of positive marker expression amongst authors and the lack of report about the staining location - if nuclear or cytoplasmic, for example. It is important to remark that due to tumor heterogeneity, sometimes the chosen tissue specimen to undergo analysis may not have a positive staining, while other samples from the same tumor might demonstrate positive results.

While much progress has been made in recent years to better understand the predictive and prognostic power of biomarkers in solid cancers, especially in the case of CD133, its utility when marking cancer stem cells is still very controversial. Additional research is necessary to develop more accurate methods of tumor profiling and thus acquire more comparable, consistently reliable results in order to better provide for patients with colorectal adenocarcinoma.

The results for CD133 demonstrate opposite data to previously described CRC studies and underline the need for its analysis in the context of other relevant molecular biomarkers and tumor/patient characteristics for reliable data interpretation regarding prognostic outcomes.

## CONCLUSIONS

CD133 expression in TMA analysis was not associated with inferior overall survival in CRC. While AXL showed inconclusive results, c-MYC expression in primary CRCs was associated with distant metastasis. 
